# A taxonomy of cognitive tasks to evaluate cognitive-motor interference on spatiotemoporal gait parameters in older people: a systematic review and meta-analysis

**DOI:** 10.1186/s11556-019-0218-1

**Published:** 2019-07-27

**Authors:** B. Wollesen, M. Wanstrath, K. S. van Schooten, K. Delbaere

**Affiliations:** 1 Human Movement Science, University of Hamburg, Mollerstr, 10, 20148 Hamburg, Germany; 2 Department for Occupational Medicine, Hazardous Substances and Public Health, German Social Accident Insurance for the Health and Welfare Services, Hamburg, Germany; 3 Neuroscience Research Australia, University of New South Wales, Sydney, Australia

**Keywords:** Falls, Concern about falls, Fear of falling, Ageing, Dual-task, Dual task cost

## Abstract

**Background:**

Walking in natural environments can be considered a dual-task (DT) scenario that requires increasing cognitive resources with advancing age. Previous reviews concluded that gait speed under DT conditions is equivalent to gait speed as a single task (ST) in the prediction of future falls in older people. However, without a clear taxonomy, these conclusions might be premature. The aim of this review is to use a taxonomy for classifying cognitive tasks of cognitive-motor interference (CMI) paradigms while walking to identify which task domains lead to more pronounced cognitive-motor decrements due to fall risk and concern about falling (CoF) in older people.

**Methods:**

A systematic literature research following PRISMA guidelines was conducted using MEDLINE, Psych-Info and EMBASE. Inclusion criteria were: older people ≥60 years with a previous fall or CoF, use of a DT paradigm to discriminate fallers and non-fallers, straight overground walking, reported gait measurements during ST and DT conditions. A meta-analysis estimated the effect of DT costs for the cognitive task domain and spatiotemporal gait parameters.

**Results:**

*N* = 3737 studies were found within the databases. Nineteen studies were included (*n* = 14 for meta-analysis). Fallers and people with CoF showed reduced walking speed for ST and DT conditions. Effects of DT were examined for mental tracking tasks. The combined odds ratio (OR [95% confidence interval]) for fallers vs. non-fallers for ST was 3.13 [0.47, 5.80] with moderate heterogeneity (*I* ^2^ = 48%). For DT, the OR was 5.17 [2.42, 7.93] with low heterogeneity (*I* ^2^ = 37%). Comparing participants with and without CoF, the OR for ST was 12.41 [9.97, 14.84] with high heterogeneity (*I* ^2^ = 85%) and OR for mental tracking DT was 10.49 [7.58, 13.40] with moderate heterogeneity (*I* ^2^ = 51%).

**Conclusion:**

CMI was not significantly different between fallers and non-fallers or people with and without CoF; however, our taxonomy revealed a large variety of cognitive conditions and a higher number of studies using mental tracking tasks, which make it impossible to draw firm conclusions. Future studies should use a more standardised and ecologically valid approach when evaluating the validity of DT gait performance in the prediction of falls, CoF or other age-related conditions.

**Trial registration:**

This review was registered at Prospero with the ID: CRD42017068912.

## Introduction

Walking in our natural environment can be considered a dual-task (DT) scenario that requires increasing cognitive resources with advancing age. Age-related decline of performance whilst walking in DT situations has been extensively investigated [1–5]. For instance, an age-related decline in gait performance has been observed when conducting arithmetic, memory or visual tasks concurrently with walking [5, 6]. Walking is not an automated task and requires structural and functional connectivity of neural brain networks. Changes in brain structure are common with ageing and require re-allocation of cognitive resources for fast and efficient operation of neural brain networks [7, 8] during complex activities. Higher age is further associated with reduced cognitive processing efficiency (e.g., decrease in nerve conduction speed and increased lateralization) [9], which is in turn associated with a decrease in cognitive performance such as diminished response time, working memory and processing of multiple tasks. These age-related cognitive changes affect daily-life task performance [10]. The level to which walking performance is affected by cognitive-motor interference is typically expressed as the dual-task cost (DTC). This is calculated as the percentage of decrements in performance in a dual- or multi-task relative to single task performance. It is proposed that, with advancing age, sensory and motor aspects of walking performance increasingly require cognitive control and attention. Several studies report a correlation between age-related declines in the sensory and motor system on the one hand and age-related declines in cognitive functioning on the other hand [11]. There is some evidence that decrements of gait performance in older people with a reduced postural reserve (motor abilities to maintain balance) can be independent of the cognitive performance [12]. Other studies showed that impaired executive function and attention affect walking performance of older fallers independent of physical ability [13, 14].

DT paradigms have become prominent to understand cognitive-motor interference (CMI) while walking in old age. These dual-task experiments have demonstrated that the extent to which the cognitive demand affects walking performance is exacerbated in old age [15], people with high risk for falls [16] and people with concerns about falling [17]. People’s tendencies to change their gait patterns during complex activities might result in an increased risk of falling [10]. Many studies reported more pronounced impairments of spatiotemporal gait parameters under dual-task conditions (including gait speed, step length, step width and double support time) in fallers compared to non-fallers [18–20]. Cognitive-motor interference in combinations with poorer physical abilities may increase a person’s risk of falling even further, especially in situations that require the adoption of a faster gait speed [21]. This is further impacted by poorer judgement of physical abilities, which has been linked to more collisions with oncoming cars in virtual reality experiments [22, 23]. The understanding of cognitive-motor interference in people with high fall risk or concerns about falling during walking under different cognitive dual-task conditions is still quite limited. Moreover, there is little information about which motor and cognitive task combinations require the highest attentional demands in older people and which mechanisms lead to insufficient resource allocation.

### Theoretical models to explain cognitive-motor interference

Several theoretical models have been proposed to explain reduced walking performance in dual-task situations. The *central bottleneck theory* states that due to an information processing bottleneck only one task can be processed at a time; processing of a second task cannot commence until the first is complete. This bottleneck usually results in a longer response time for one of the two tasks [34–36]. The *4-dimensional multiple resource model* [37] proposes that there will be greater interference between two tasks that utilise similar resources. Finally, the *attentional resource theory* suggests that declines in performance under DT conditions result from interference caused by competing demands for attentional resources, resulting in less attention available to each task [38, 39].

The *attentional resource theory* might especially apply to people with CoF. CoF is very common in older people and can lead to self-induced restriction of physical and social activities. In its most severe form, it can result in a persistent and dysfunctional disruption of attention. People with higher levels of CoF have difficulties to inhibit or ignore irrelevant information of the environment in the process of balance control. Therefore, CoF may compete for the limited resources of attentional focus to maintain balance control during complex activities [40] resulting in instability and increased fall risk. A meta-analysis by Ayoubi et al. [41] revealed that CoF is associated with increased gait variability during normal walking. This effect is amplified under DT conditions, due to reduced gait speed and step length (often referred to as cautious gait), especially in older people who also reduce their daily physical activity due to their CoF [42].

Performance is expected to deteriorate in complex situations if there are fewer resources available for performance than are required. Navon [43] defined resources as any internal input that is essential for processing and is available in limited quantities at any point in time. Walking requires coordination of peripheral sensory and neuromuscular systems, with higher-level cognitive processing, which gradually decline with age. It is therefore not surprising that with advancing age, cognitive-motor interference becomes more pronounced when performing complex daily activities [10, 36, 44]. Each task requires a reweighting of sensorimotor information depending on the requirements of the additional task [45]. When the sensory system delivers conflicting information, vision will dominate spatial processing, which impacts a person’s ability to coordinate sensory and cognitive processing to main upright [45]. In addition, studies indicate that increasing difficulty levels (from DT to multitask-performance or with different task complexities e.g. from processing speed to decision-making tasks; see Table 1) further amplify the effects of cognitive-motor interference on walking performance [46–51]. Systematic reviews have further highlighted that cognitive-motor interference rises based on the task domain and the individual’s abilities and resources [52, 53]. More specifically, tasks including controlled processes or motor components showed more decrements in DT performance of older people.

**Table 1:** Proposed taxonomy for cognitive dual tasks

A range of DT paradigms have been used to examine age-related changes in motor performance and cognitive capacities, and to understand the relationship between performance in motor and cognitive tasks [24]. Cognitive tasks can be classified according to their demands and the mental processes involved to execute them. Based on the most commonly used tasks in DT-studies, the following domains were defined; each domain is distinct from the other domains at a behavioral and/or cognitive level according to definitions of Colcombe and Kramer [25] and Al-Yahya et al. [1]:	
• Reaction time tasks (processing speed) refer to tasks that involve the measurement of elapsed time between a sensory stimulus and a behavioral response [26]. These tasks are used to measure processing speed, where a slowed processing might underlie an attentional deficit [27]; e.g., a simple reaction time test to press a button following a light stimulus.	
• Controlled processing tasks refer to tasks that involve decision making in addition to processing speed; e.g., to press a button when a star is presented on a screen.	
• Visuospatial tasks refer to task that require detecting or processing visual information; e.g. Benton Visual retention task [28] or naming the location of a stimulus.	
• Mental tracking tasks refer to tasks that require holding information in the mind while performing a mental process [27]. These tasks have been usually used to examine sustained attention and information processing speed [27, 29]. The most common mental tracking tasks are:	
a. Arithmetic tasks refer to tasks that require solving a mathematical equation or counting backwards in threes or sevens.	
b. Verbal fluency tasks refer to tasks that require word production, either spontaneously or under pre-specified search conditions; these tasks have recently been used to examine executive functions [26, 27].	
• Working memory tasks refer to tasks that require holding information in the mind which is available for processing [30]. The differentiation between working memory and mental tracking tasks was adapted from brain imaging studies [31]. Tasks that require holding information only, are categorized into working memory tasks, while those that require holding information plus manipulation belong to the mental tracking category [31, 32]; e.g., a n-back task.	
• Discrimination tasks refer to tasks that require selective attention to a specific stimulus or feature and respond accordingly; they have been usually used to examine attention and response inhibition such as the Stroop paradigm [33] or a Go or no-go task.	

However, activities that heavily rely on postural control occasionally lead to an improved motor performance when combined with a secondary task [54]. The *U-Shaped Non-Linear Interaction Model* postulates that, depending on the complexity of the secondary task, motor and balance performance can increase or decrease [55]. For example, there might be a reduction of postural sway as a result of muscle co-contraction while concentrating on the cognitive task [56, 57], whereas postural sway may increase without additional cognitive performance with a secondary task [58]. The *Supra-Postural Task Model* [59, 60] provides additional details to explain the U-shape relationship between postural control and balance. The theory suggests that in specific situations the motor performance is necessary to reach the goal of the cognitive task (e.g. standing still to read a sign). In contrast to the *U-shaped model,* in the *Supra-Postural Task Model* effects are explained by situation awareness and not by task complexity [61].

Finally, the *Task Prioritization Model* [62] accounts for the strategies that an individual might use during complex activities. It postulates that older people are more likely to prioritize motor performance under threat of a loss of balance [63, 64]. This prioritization reduces the cognitive-motor interference and allows for reorganization of the cognitive-motor resources [65] to reduce the risk of falling. However, if the environment poses too many challenges (e.g. elevated surface), task prioritization is not always effective. Yogev-Seligmann and colleagues [66] found that older people with adequate balance abilities and capacity to identify hazards are able to focus on cognitive performance as long as balance is maintained. On the other hand, fallers are not able to shift attention in these situations [67], which could be explained by the impact of poor executive function and attention on walking performance of older fallers [13, 14].

### Objectives

The primary objective of this review was to use a taxonomy for classifying cognitive tasks to gain insight in cognitive-motor interference within the study of falls in older people. Previous reviews concluded that gait speed under DT conditions is equivalent to gait speed as a single task in the prediction of future falls in older people [50, 68]. However, without a clear taxonomy of cognitive dual tasks, these conclusions might be premature. In addition, little is known about the effects of dual-task settings on older adults with CoF. A clear taxonomy will allow a better understanding of how cognitive-motor interference during complex activities is related to fall risk and concern about falls.

## Methods

### Search strategy

Databases were systematically searched by using OvidSp to search in Medline (1946 to 2019, Week 20), Embase (1974 to 2019, Week 20) and PsycINFO (1806 to 2019, Week 20). The search within the databases was limited to the English and German language. In addition, the reference lists of included articles were searched manually. Two reviewers (BW, MW) independently searched within titles and abstracts to identify all potentially eligible studies. Afterwards, these two reviewers independently assessed full paper copies of the identified potentially eligible studies to determine the studies to be included. Any disagreement on inclusion was resolved by discussion and through arbitration by a third reviewer (KvS, KD).

### Inclusion and exclusion criteria

The inclusion criteria were: (i) older adults ≥ mean age of the sample was 60 years with a previous fall or CoF, (ii) the dual-task paradigm was used to discriminate fallers from non-fallers or people with high concerns about falling from people with low concerns about falling, (iii) utilized straight over ground walking at self-selected speed as the primary motor task, (iv) reported gait measurements during both single and dual-task performance, or the effect of dual-tasking on gait performance (more than one gait cycle), (v) clear description of the dual-task situation, (vi) reported adequate data to calculate effect sizes either from descriptive or inferential statistics, (vii) interventional studies were included if the effect of dual-tasking on gait at baseline was reported. The exclusion criteria included: (i) population with brain injuries or diagnosed cognitive decline, (ii) physical impairments (e.g. using a cane or walker) and (iii) chronic diseases (e.g., multiple sclerosis or Parkinson’s disease). Moreover, studies with a secondary analysis of previous reported results were also excluded.

### Selection criteria

Studies comparing fallers and non-fallers were included if the method section reported of the number of falls. Prospective studies were considered if they compared fallers and non-fallers at baseline (retrospective) or at the follow-up measurement und ST and DT conditions.

Studies addressing CoF were included if they classified the participants according to the “falls efficacy scale international (FES-I)” [69] score, the activities-specific balance confidence (ABC) scale [70] or if they asked the participants using a single item question if they were afraid of falling during activities of daily life.

Studies that included walking under DT conditions were included. This includes studies that investigated at least one walking task (in a DT setting; according to the definitions of spatiotemporal gait parameters addressed in Table 2), studies that compare ST and DT performance, and studies that investigated DT performance in healthy or balance-impaired (fallers) older adults in either a randomized control trail (RCT), an experimental-control group design or an old-young comparison. Moreover, studies with a secondary motor task were also included. Additionally, every concurrent task was assigned to a “stimulus-response-condition” (visual-verbal, visual-manual, auditory-verbal, auditory-manual) and classified according to our taxonomy of cognitive tasks (see Table 1).

**Table 2:** Spatiotemporal gait parameters

Gait – the medical term used to describe the locomotor movement of walking – is simple in terms of execution, but complex in terms of biomechanics and motor control [73]. During steady-state straight forward gait, commonly examined gait variables can be classified into parameters of rhythm (e.g. single and double support time or cadence) and pace (e.g. speed or stride length). This review follows the Guidelines for Assessment of Gait and Reference Values for Spatiotemporal Gait Parameters in Older Adults [73] by defining spatiotemporal gait parameters as:	
• Stride length: a stride is the distance from heel strike of one extremity to the next heel strike of the same extremity. Stride length is the distance that one part of a foot travels between the same instant in two consecutive gait cycles.	
• Step length: step length is the distance that one part of the foot travels in front of the same part of the other foot during each step.	
• Cadence: measure of the number of steps per unit time. Cadence increases if step length shortens when gait speed is held constant.	
• Walking speed: distance travelled divided by the ambulation time. Speed was expressed in centimetres per second (cm/sec).	
• Double support time: amount of time spent with both feet in contact with the ground. The gait cycle is divided into the stance phase, when the foot is in contact with the floor, and the swing phase, when it is not. The double support time is approximately 20% of the gait cycle during which both feet are in ground contact.	

### Quality assessment

Quality assessment of the included articles was based on the Standard Quality Assessment Criteria (SQAC) for evaluating primary research papers proposed by the Alberta Heritage Foundation for Medical Research [71]. As the review did not focus on RCTs, the quality criteria for RCTs were not assessed. The quality criteria, as described in SQAC, were: (1) sufficient description of the question/objective; (2) appropriate study design; (3) appropriate method of participant selection or source of information/ input variables; (4) sufficient description of participant characteristics; (5) report of means of assessment with outcome measures well defined and robust" to measurement or misclassification bias (6) appropriate sample size; (7) appropriate analytic methods and method description; (8) report of estimate of variance in main results; (9) control for confounding; (10) sufficiently detailed report of results; and (11) conclusions supported by the results.

Participant selection was verified by comparing the sample with the conclusions drawn from the experimental results. A full point for appropriate sample size was given when either an a priori calculation of sample size had been described or the sample size was a full cohort. Based on the analytic methods employed (8), important statistical values (according to the APA-Manual [72]) had to be included to obtain a full quality score. BW and MW or KvS performed the assessment independently and the results presented in Table 3 were concurred on. Each criterion scored one point if partly fulfilled and two points if completely fulfilled. Points were added up and resulted in the quality score. The necessary score for a study of high quality was defined to be 17 out of 22 (75%) and 10–16 points for standard quality according to the SQAC. No point was given if general remarks had to be made (indicated by brackets; Table 3). Moreover, we reported some general methodological issues (cf. column general marks). Studies were included in the meta-analysis if they had quality score of 7 or more.

**Table 3:** Quality score

Author	Year	1	2	3	4	5	6	7	8	9	10	11	Quality score	Remarks
Asai [87]	2014	(x)	(x)	x	x	(x)	x	x	x	no	x	x	17	design is not clearly identified; differences in subgroups variables sex (f/m), no studies for accuracy of accelerometer, SEM or validity
Auvinet [74]	2017	x	x	x	x	(x)	(x)	(x)	x	(x)	x	(x)	17	no studies for accuracy of equipment, SEM or validity; study is mainly descriptive: no control for baseline characteristics; sample size for subgroup “cautious gait” (< 10) low
Bauer [75]	2010	(x)	(x)	x	(x)	x	x	x	(x)	no	x	x	16	design is not clearly identified
Beauchet [19]	2008	x	(x)	x	x	no	x	x	x	no	x	x	17	design is not clearly identified; stopwatch as measurement
Bootsma-van der Wiel [76]	2003	x	x	x	(x)	no	x	x	(x)	x	(x)	x	17	no description of BMI or weight; no report of variance; stopwatch as primary measurement tool
Donoghue [88]	2013	x	x	x	x	x	x	x	no	no	x	x	18	no reporting of estimate variance in results and confounding
Freire Junior [21]	2017	x	(x)	(x)	(x)	x	x	x	no	x	x	x	17	design is not clearly identified; no sufficient description of the population group; no medications or comorbidities reported
Howcroft [77]	2016	x	(x)	x	no	x	(x)	x	no	no	x	x	14	no gait specifications: design is not clearly identified; no description of BMI, cognition or number of medications, no baseline comparison reported
Johannsson [78]	2016	(x)	x	(x)	x	x	x	(x)	x	x	x	x	19	no mention of clear gait parameters (“challenging gait conditions”); research question and hypothesis missing; no sufficient information; in the method section
Mirelman [79]	2012	x	(x)	x	x	x	x	x	x	no	x	x	19	design is not clearly identified
Muhaidat [20]	2013	x	(x)	x	(x)	no	(x)	x	no	(x)	x	x	14	design is not clearly identified; no description number of medications/drugs or disease; stopwatch used as primary measurement tool; pilot study with small sample size (no sample size calculation); differences of baseline characteristics reported but no statistical analysis done
Nordin [80]	2010	x	x	x	x	x	x	x	no	no	(x)	x	17	only medians reported
Reelick [81]	2011	(x)	(x)	(x)	x	x	(x)	(x)	x	no	x	x	15	no gait specifications; design is not clearly identified, no description of BMI or height; small sample size
Reelick [89]	2009	x	(x)	(x)	x	x	(x)	(x)	x	no	x	x	16	design is not clearly identified, small sample size
Springer [82]	2006	x	(x)	(x)	(x)	(x)	(x)	x	(x)	x	(x)	x	15	no sufficient information about study design; no description number of medications/drugs or disease
Toulotte [83]	2006	(x)	x	x	(x)	x	(x)	x	x	x	x	x	19	research question is not precise; no description number of global cognition and medication use or diseases
Toulotte [84]	2006	x	(x)	x	(x)	x	(x)	(x)	x	x	x	x	18	No description of global cognition; report of means and estimate of variance in main results are missing
Verghese [85]	2017	(x)	(x)	x	(x)	x	x	x	(x)	no	(x)	x	15	research question is unclear; study design unclear; no report of variance
Wollesen [90]	2017	x	x	x	x	x	x	x	x	x	x	x	22	
Yamada [86]	2011	(x)	(x)	no	(x)	(x)	no	(x)	no	no	no	no	5	research question and hypothesis missing; insufficient information of the other items

Legend: x: yes, (x): partially with general remarks, no: no/unclear. Item 1, sufficient description of question/objective; 2, appropriate study design; 3, appropriate method of participant selection or source of information/input variables; 4, sufficient description of patient characteristics; 5, report of means of assessment with outcome measures well defined and robust to measurement/misclassification bias; 6, appropriate sample size; 7, appropriate analytic methods and method description; 8, report of estimate of variance in main results; 9, control for confounding; 10, sufficiently detailed report of results; 11 conclusions supported by the results. Specifications: 1: clearly description, mention of specific gait parameters (no generalization e.g. gait changes), 2 / 3: precise information of in- and exclusion criteria and recruitment, 4: specific baseline characteristics of population (older people) must include: age, distribution male/female, global cognition, BMI or height/weight, number of medications or chronic disease/comorbidities, and for healthy older people: TuG, SPPB or comparable test of physical abilities, 5: Primary and secondary outcome must be clearly described. Error of measurement must be discussed, 6: sample size calculation should be reported 8: Means and estimate of variance (SD or IQR) must be reported for main outcome

### Data extraction

Table 4 provides an overview of all included studies including the authors, year of publication, study design and aims, population with discrimination to fallers/non-fallers or participants with concerns or no CoF, observed walking parameters and description of the DT setting. The main results of the studies were extracted to Table 5. This includes task order, outcome measures used to assess and report the concurrent tasks performance and instructions given to participants, and study results. Data were recorded as a mean and standard deviation (SD) if reported, with sample size and number analyses in each group (fallers vs. non-fallers or participants with concerns or no CoF).

**Table 4:** Included studies with fallers

Author	Title	Study design	Study aims	No. of subjects	Age (yr)	Dual-task type
Auvinet et al., 2017 [74]	Gait disorders in the elderly and dual task gait analysis	Prospec-tive cohort study	1. To assess the value of gait instability as a clinical symptom, 2. To quantify gait disorders by means of the DTC in order to differentiate between peripheral pathologies and central nervous system pathologies, 3. To identify motor phenotypes according to the DTC for stride frequency and gait regularity 4. To identify correlations between these motor phenotypes and conventional brain MRI findings.	Overall (*n* = 103), Gait instability (*n* = 46), Recurrent falls (*N* = 30), Memory impairment (*n* = 19), Cautious Gait (*n* = 8) Falls assessment of previous falls in the last 12 months	Overall (76 ± 7), Gait instability (77 ± 8), Recurrent falls (77 ± 8), Memory impairment (76 ± 5), Cautious Gait (81 ± 5)	Arithmetic DT: Walking + counting aloud backwards from 50 subtracting serial 1 s (one by one) SR: auditory-verbal
Bauer et al., 2010 [75]	First Results of Evaluation of a Falls Clinic	Cross-sectional study	To assess risk factors for falls in community dwelling older people and to recommend targeted interventions	Fallers (*n* = 42) Non-fallers (*n* = 19) Previous falls in the last 12 months were assessed by questionnaire	Fallers (75.95) Non-fallers (75.35) No SD reported	Arithmetic DT: 1. Walking+ counting backwards from 50 subtracting serial 1 s (one by one) 2. walking + subtracting serial 3 s from 100 Verbal fluency task: Walking + naming animals SR: auditory verbal
Beauchet et al., 2008 [19]	Recurrent falls and dual task-related decrease in walking speed: is there a relationship?	Prospec-tive cohort study	To determine whether DT–related changes in walking speed were associated with recurrent falls in frail older people	Fallers (*n* = 37) vs Non-Fallers (*n* = 156) vs Recurrent fallers (*n* = 20) Falls assessment of previous falls in the last 12 months	Fallers (84.7 ± 5.1) vs Non-Fallers (83.9 ± 5.5) vs. Recurrent fallers (87.2 ± 5.7)	Arithmetic DT: Walking + subtracting serial 1 s (one by one) from 50 SR: auditory verbal
Bootsma-van der Wiel et al., 2003 [76]	Walking and talking as predictors of falls in the general population	Prospective population-based follow-up study	To compare the added value of DT in predicting falling in the general population of oldest old with that of an easy-to-administer ST	None (*n* = 22), One (*n* = 87), and Recurrent (*n* = 71) Previous falls in the last 12 months and the last month assessed by questionnaire	Leiden 85-plus Study (no precise information)	Verbal fluency task: Walking + reciting names of animals or professions during a 30-s period SR: auditory verbal
Freire Junior et al., 2017 [21]	The effects of a simultaneous cognitive or motor task on the kinematics of walking in older fallers and non-fallers	Cohort study	Comparing kinematics of ST gait, cognitive DT gait, and motor DT gait in both older fallers and non-fallers	No Falls (*n* = 35) vs. One fall (*n* = 27) Previous falls in the last 6 months and the last month assessed by questionnaire	No Falls (67.97 ± 4.82) vs One fall (67.96 ± 5.7)	1. Verbal fluency task: Walking + naming animals SR: auditory verbal 2. Secondary motor task: Walking + transferring a coin from one pocket to another SR: auditory-manual
Howcroft et al., 2016 [77]	Analysis of dual-task elderly gait in fallers and non-fallers using wearable sensors	Prospective population-based follow-up study	Use wearable sensors to detect gait differences between: (1) fallers and non-fallers for ST walking, (2) between fallers and non-fallers for DT walking, (3) ST and DT walking for fallers, and (4) ST and DT walking for non-fallers.	Fallers (*n* = 24), Non-Fallers (*n* = 76) Previous falls in the last 6 months and the last month assessed by questionnaire	Fallers (76.3 ± 7,0), Non-Fallers (75,2 ± 6,6)	Verbal fluency task: Walking + naming words starting with A, F or S) SR: auditory verbal
Johansson et al., 2016 [78]	Greater Fall Risk in Elderly Women Than in Men Is Associated With Increased Gait Variability During Multitasking	Prospective observational study	1. investigate variability in gait patterns among men and women aged 70 years during progressively challenging gait conditions. 2. investigate associations of gender with gait patterns and the risk of incident falls.	Non-Fallers (1202), Fallers (148) Falls assessment of previous falls in the last six and 12 months	Fallers (70), Non. Fallers (70), Inclusion criteria: age of exactly 70 years at the time of testing.	Arithmetic task: Walking + subtracting 1 s from the number 100 SR: auditory verbal
Mirelman et al., 2012 [79]	Executive function and falls in older adults	Cohort study (Follow Up)	(1) Evaluate if reduced Executive Function is a risk factor for future falls, (2) assess whether ST and DT walking abilities, an alternative window into EF, were associated with fall risk.	256 Participant recorded there falls in a falls calendar	76.4 ± 4.5	Arithmetic task Walking + subtracting 3 s from a predefined 3 digit number SR: auditory verbal
Muhaidat et al., 2013 [20]	Exploring gait-related dual task tests in community-dwelling fallers and non-faller	Pilot study	Assess differences in DT performance between the two groups on both primary and secondary tasks to help narrow down the potential choices of tasks, using simple clinical outcome measures that only require the use of a stopwatch, for future research.	Fallers (*n* = 12), Non-Fallers (*n* = 15) Falls assessment of previous falls in the last 12 months	Fallers (75.5 (8.5 IQR), Non-Fallers 72 (4 IQR)	Arithmetic tasks: 1. Walking + subtracting 3 s 2. Walking + subtracting 7 s Verbal fluency tasks: 3. Walking + Generating words e starting with the letter I, N, or O 4. Walking + Generating animal names Visual spatial task: 5. Walking + clock task SR 1.-5.: auditory verbal Secondary motor task: 6. Walking + carrying a cup SR: auditory- manual Discrimination task: 7. Walking + Stroop (high/ low; different pitches) SR: auditory verbal
Nordin et al., 2010 [80]	Changes in step-width during dual-task walking predicts falls	Cohort study (Follow Up)	Evaluate whether gait pattern changes between single- and DT conditions were associated with risk of falling in older people	Fallers (*n* = 120), Non-Fallers (*n* = 110) Participant recorded there falls in a falls dairy	Non-Fallers (78) Fallers (80) No SD available	1. Secondary motor task: Walking + carrying a cup on a tray) SR: auditory-manual 2. Verbal fluency task: Walking + naming animals 3. Arithmetic task: Walking + subtracting 3 s from 50 SR 2. + 3.: auditory verbal
Reelick et al., 2011 [81]	Increased intra-individual variability in stride length and reaction time in recurrent older fallers	Cross-sectional study	To compare mean performance measures as well as intra-individual variability measures of stride length and reaction time in vulnerable recurrent and non-recurrent older fallers.	Non- fallers (*n* = 38), Recurrent fallers (*n* = 22) Falls assessment of previous falls in the last 6 months	Non-recurrent Fallers (75.8 ± 7.2), recurrent Fallers (75.7 ± 5.6)	1. Arithmetic task Walking + subtracting 7 from 100 2. Verbal fluency task: Walking + naming words starting with a certain letter SR: auditory verbal
Springer et al., 2006 [82]	Dual-tasking effects on gait variability: The role of aging, falls, and executive function	Cross-sectional study	Test if the DT effect on gait variability is larger 1) in healthy older people vs healthy young people; 2) in idiopathic older fallers vs healthy older people; 3) and if DT has effects on gait variability are correlated with executive function	YA (*n* = 19), Non- fallers (*n* = 24), Fallers (*n* = 17) Falls assessment of previous falls in the last 6 months	YA (29,4 ± 4,4), Non-Fallers (71.0 ± 5.9), Fallers (76.1 ± 4.8)	1. Listening memory task Walking + listening to a text; answering questions afterwards SR: auditory verbal 2. Arithmetic task Walking + subtracting 7 from 500 SR: auditory verbal
Toulotte et al., 2006a [83]	Effects of training and detraining on the static and dynamic balance in elderly fallers and non-fallers	Pilot study	Evaluate the effects of training on static and dynamic balance in ST and DT conditions to analyze the effects of detraining on static and dynamic balance in healthy older fallers and non-fallers.	Fallers (*n* = 8), Non-Fallers (*n* = 8) Falls assessment of previous falls in the last 24 months	Fallers (71.1 ± 5.0), Non-Fallers (68.4 ± 4.5)	Secondary motor task: Walking + carrying a glass SR: auditory-manual
Toulotte et al., 2006b [84]	Identification of healthy elderly fallers and non-fallers by gait analysis under dual-task conditions	Case comp-arison study	Compare healthy older fallers and non-fallers to identify balance disorders associated with falling under ST and DT conditions	Fallers (*n* = 21), Non-Fallers (*n* = 19) Falls assessment of previous falls in the last 24 months	Fallers (70.43 ± 6.43), Non-Fallers (67.05 ± 4.81)	Secondary motor task: Walking + carrying a glass SR: auditory-manual
Verghese et al., 2017 [85]	Brain activation in high-functioning older adults and falls	Prospective cohort study	To determine whether brain activity over the prefrontal cortex measured in real time during walking predicts falls in high-functioning older people	166 71 fallers 95 non-fallers Falls were prospectively ascertained over a 50-month period	74.95 ± 6.07	Letter memory task: Walking + reciting alternate letters SR: auditory-manual
Yamada et al., 2011 [86]	The reliability and preliminary validity of game-based fall risk assessment in community-dwelling older adults	Randomized controlled trial	Examine whether the Nintendo Wii Fit program could be used for fall risk assessment in healthy, community-dwelling older people	Faller (*n* = 16), Non-Fallers (*n* = 28) Previous falls in the last 12 months assessed by question-naire	Fallers (84.8 ± 10.1), Non-Fallers (80.2 ± 6.4)	Arithmetic task: Walking + counting backwards from 50 SR: auditory-manual
Asai et al. 2014 [87]	Effects of dual-tasking on control of trunk movement during gait: respective effect of manual- and cognitive-task	Cross-sectional study	1. to assess the effects of a cognitive task and a manual task on trunk movements during gait. 2. to examine the effect of FoF on trunk movement in both dual-task walking conditions: cognitive task and manual-task gaits.	Overall (*n* = 117), nFoF (*n* = 85), FoF (*n* = 32) Previous falls in the last 12 months assessed by questionnaire FOF was assessed by one question	Overall (73.7 ± 4.0), nFoF (73.7 ± 4.0), FoF (74.5 ± 4.0)	1. Arithmetic task: Walking + subtracting 1 s from 100 SR: auditory-manual 2. Secondary motor task: Walking + motor task carrying a ball on a tray SR: auditory-manual
Donoghue et al., 2013 [88]	Effects of fear of falling and activity restriction on normal and dual task walking in community dwelling older adults	Prospective cohort study	1. to examine the relationship between FOF, activity restriction and gait characteristics in normal and dual task walking and 2. to determine if these relationships persist after adjusting for potentially underlying factors	No FOF (*n* = 961), FOF-NAR (*n* = 250), FOF-AR (*n* = 96) FOF was assessed by one question	No FOF (72.3 ± 5.6), FOF-NAR (74.9 ± 5.8), FOF-AR (73.9 ± 5.6)	Verbal memory task: Walking + recite alternate letters of the alphabet (A-C-E, etc.) SR: auditory-manual
Reelick et al., 2009 [89]	The influence of fear of falling on gait and balance in older people	Cross-sectional study	The purpose of this study was to examine the association between FoF and gait and balance in older people during walking with and without dual-tasking.	FoF (*n* = 29) vs NFOF (*n* = 65) FOF was assessed by the ABC-NL scale	FOF (80.6 ± 4.2) vs. NFOF (80.5 ± 3.7)	Arithmetic task: Walking + subtracting 7 s from 100 SR: auditory-manual
Wollesen et al., 2017 [90]	Does dual task training improve walking performance of older adults with concern of falling?	Single blind randomized controlled trial	The primary aim of this study was to compare the effects of a DT training integrating task managing strategies for independent living older people with and without concern about falling to a non-training control group on walking performance under ST and DT conditions.	Intervention with FES-I < 20 (*n* = 26) vs Intervention with FES-I > 20 (*n* = 30) vs Control group with FES-I < 20 (*n* = 19) vs Control group with FES-I (*n* = 20)	Intervention with FES-I < 20 (72.2 ± 4.6) vs Intervention with FES-I > 20 (69.8 ± 5.7) vs Control group with FES-I < 20 (72.9 ± 4.4) vs Control group with FES-I (72.7 ± 5.3)	Discrimination task: Walking + Stroop task (colors) SR: visual-verbal

Legend: *SR* Stimulus-reponse condition, *YA* Young adults, *nFOF* No fear of falling, *FOF* Fear of falling, *FOF NAR*=, *ABC-NL* Advanced balance scale Netherlands, *FES-I* Falls efficacy scale- international

**Table 5:** Data extraction fallers/ non-fallers

Author	Dual-task category	Stimulus-Response condition	Recording of the gait parameters	Gait parameters Single & Dual Task	Results
Auvinet et al., 2017 [74]	walking+ arithmetic (counting backwards from 50 subtracting serial 1 s)	auditory -verbal	3-D-acceleration sensor, Locometrix (electronic walkway), stopwatch, 30 (m) distance	walking speed (m/s) stride frequency (Hz) and the stride regularity (dimensionless)	1. Non-Fallers: walking speed (m/s) ↓, stride frequency (Hz) ↓ and the stride regularity (dimensionless) ↓ 2. Fallers: walking speed (m/s) ↓, stride frequency (Hz) ↓ and the stride regularity (dimensionless) ↓ Walking speed and stride regularity differed between subgroups under ST and DT (*p* < 0.01 and 0.05, respectively, for both conditions).
Beauchet et al., 2008 [19, 96]	walking+ arithmetic (counting backward (counting backwards from 50 subtracting serial 1 s)	auditory -verbal	Stopwatch, 10 (m)	walking speed (cm/s), cadence (steps/min)	1. Non-Fallers (0 falls): walking speed (cm/s) ↓, cadence (steps/min) ↓ 2. Non-multiple Fallers (0 or 1 fall): walking speed (cm/s) ↓, cadence (steps/min) ↓ 3. Fallers (1 or more falls): walking speed (cm/s) ↓, cadence (steps/min) ↓ 4. Multiple Fallers (2 or more falls (walking speed (cm/s) ↓, cadence (steps/min) ↓ Single and recurrent fallers walked more slowly than non-fallers under ST and DT conditions
Bauer et al. 2010 [75]	walking+ arithmetic (counting backwards from 50 subtracting serial 1 s) walking + arithmetic (counting backwards from 100 subtracting serial 3 s) walking + verbal fluency (naming animals starting with a specific letter)	auditory verbal	GAITRite system	walking speed (cm/s)	Fallers walked slower than non-fallers under ST and DT conditions. Both non-fallers and fallers showed comparable reduced DT performance for the task conditions. The verbal fluency task had a higher amount of DCT in comparison to the arithmetic task (cf. Fig. 5)
Bootsma et al., 2003 [76]	walking + verbal fluency (reciting names of animals or professions during a 30-s period)	auditory -verbal	Stopwatch, 12 (m)	walking time (s) Number of steps (n), Number of complete stops (n)	1. Non-Fallers (0 falls): walking time (s) ↑, Number of steps (n) ↑ 2. non-multiple Faller (0 or 1 fall): walking time (s) ↑, Number of steps (n) ↑ 3. Fallers (1 or more falls): walking time (s) ↑, Number of steps (n) ↑
Freire Junior et al., 2017 [21]	1. walking + word task (naming animals starting with a specific letter) 2. walking + motor task (transferring a coin from one pocket to another)	1. auditory - verbal 2. manual	GAITRite, 8(m), 25–36 steps were collected to examine variability	gait speed (m/s), cadence (steps/min), step length (cm) stride time (s), single support time (as percentage of the gait cycle), and stride time variability (CoV (%))	1. Non-Fallers (0 falls) and Fallers (1 or more falls) + word task: gait speed (m/s) ↓, cadence (steps/min) ↓, stride time (s) ↑, step length (cm) ↓, single support time ↓, and stride time variability (CoV (%))↑ 2. Non-Fallers (0 falls) and Fallers (1 or more falls) + motor task: gait speed (m/s) ↓, cadence (steps/min) ↓, stride time (s) ↑, step length (cm) ↓, single support time (↓, and stride time variability (CoV (%)) ↑ There were no significant main effects of group and interaction effects between group and task.
Howcroft et al., 2016 [77]	walking + word fluency task (recite words starting with A, F or S)	auditory – verbal	Stopwatch, Pressure-sensing insoles (F-Scan 3000E, Tekscan, Boston, MA), Accelerometers (X16-1C, Gulf Coast Data Concepts, Waveland, MS), 7,62 (m)	cadence (steps/min), double support time (%), speed (m/s) CoP path (s), Min CoP Vel (m/s), Mean Cop Vel (m/s), Median CoP Vel (m/s), Cadence (steps/min), stride time (s), stance time (s), swing time (s), stride time CoV, percent stance time (%), stride time symmetry index, Impulse (Foot-strike to first peak (Ns/kg), Min to second peak (Ns/kg), Second peak to foot-off (Ns/kg), Foot strike (Ns/kg)	1. Non-Fallers (0 falls): cadence (steps/min) ↓, Percent double support time (%) ↓, gait speed (m/s) ↓ 2. Non-multiple fallers (0 or one falls): cadence (steps/min) ↓, Percent double support time (%) ➔ no data, gait speed (m/s) ↓ 3. Fallers (1 or more falls): cadence (steps/min) ↓, Percent double support time (%) ↓, gait speed (m/s) ↓ 4. Multiple Fallers (2 or more falls): cadence (steps/min) ↓, Percent double support time (%) ➔ no data, gait speed (m/s) ↓ Accelerometer: Differences were found between fallers and non-fallers for the head and posterior pelvis accelerometers.
Johansson et al., 2016 [78]	walking+ arithmetic task (counting backwards from 10 by subtracting serial 1 s)	auditory – verbal	GAITRite, 8.6 (m)	Double support time CV(%), gait speed (m/s) at baseline (normal speed) -- > All gait parameters present as Coefficient of Variations (CVs; SD/M × 100)	For Non-Fallers (0 falls) and Fallers (1 or more falls): step width CV (%) ↑, stride width CV (%) ↑, step length CV (%) ↑, stride length CV (%) ↑, step time CV (%) ↑, stance time CV (%) ↑, stride time CV (%) ↑, stride velocity CV (%) ↑, swing time CV(%) ↑, DST CV(%) ↑
Mirelman et al., 2012 [79]	walking + arithmetic task (counting backwards from a predefined 3 digit number by subtracting serial 3 s)	auditory - verbal	force-sensitive insoles, stopwatch, 25 (m)	gait speed (m/s) gait variability (%)	1. Non-fallers (0 falls), Non-multiple fallers (1 fall), Multiple fallers (2 or more falls): gait speed (m/s) ↓
Muhaidat et al., 2013 [20]	1. walking + arithmetic task (counting backwards by subtracting serial 3 s/ 7 s) 2. walking + Stroop task (identify incongruency between the words high/low in different pitches) 3. walking + word task (generating words starting with I, N, O or naming animals) 4. walking + visuospatial task (clock task) 5. walking + motor task (carrying a cup)	1. auditory-verbal 2. auditory -verbal 3. auditory -verbal 4. visual-spatial (manual) 5. manual	stopwatch, 10 (m)	Gait speed (m/s)	Gait speed (m/s) ↓ for all subgroup form ST to DT A trend of a difference in complexity of secondary and absolute values of task performance was shown between community-dwelling fallers and non-fallers (*p* ≤ 0.05).
Nordin et al., 2010 [80]	1. walking + motor task (carrying a cup) 2. walking + motor task (carrying a tray) 3. walking + word/verbal fluency task (animals naming) 4. walking + arithmetic task (counting backwards from 50 by subtracting serial 3 s)	1. manual 2. auditory -verbal 3. auditory -verbal	GAITRite, 10 (m)	walking speed (m/s), step length (cm), double support time (ms) step width (mm), step time (ms)	1a. Non-fallers (0 falls) and Fallers (1 or more falls) + motor task (cup): walking speed (m/s) ↑ 1b. Non-fallers (0 falls) and Fallers (1 or more falls) + motor task (tray): walking speed (m/s) ↑ 1c. Non-fallers (0 falls) and Fallers (1 or more falls) + word task: walking speed (m/s) ↓ 1d. Non-fallers (0 falls) and Fallers (1 or more falls) + arithmetic task: walking speed (m/s) ↓
Reelick et al., 2011 [81]	1. walking + arithmetic task (counting backwards from 100 by subtracting serial 7 s) 2. walking + word task (naming words starting with a given letter)	1. auditory -verbal 2. auditory -verbal	GAITRite, 6.1 (m)	gait velocity (cm/s), stride length (cm) Number of strides (n), stride length CV (%), stride time (cm), stride time CV, stride width (cm), stride with CV (%)	1. Non-multiple Fallers (0 or 1 fall) and Multiple Fallers (zer0o or 1 fall) + arithmetic task: gait velocity (cm/s) ↓, Number of strides (n) ↑, stride length (cm) ↓, stride length CV (%) ↑ 2. Non-multiple Fallers (0 or 1 fall) and Multiple Fallers (0 or 1 fall) + word task: gait velocity (cm/s) ↓, Number of strides (n) ↑, stride length (cm) ↓, stride length CV (%) ↑ Stride-length CV was higher when participants performed a DT, and higher in recurrent fallers compared with non-recurrent fallers, although this difference was only significant during performance of the verbal fluency task
Springer et al., 2006 [82]	1. walking + listening 2. walking + listening plus answering questions3. walking + arithmetic task (counting backwards from 500 by subtracting serials 7 s)	1. auditory -verbal2. auditory -verbal	force-sensitive insoles, 25 (m)	gait speed (m/sec) ➔ Gait speed was normalized with height, average swing time (%) swing time variability CV (%)	In swing time variability fallers and elderly non-fallers differed (*p* = 0.003)
Toulotte et al., 2006a [83]	walking + motor task (carrying a glass)	manual	VICON 370 system (Oxford Metrics), Three AMTI force plates (250 Hz), 10 (m)	cadence (steps/min), walking speed (m/s), stride length (m) stride time (s), step time (s), single-support time (s)	1. Non-Fallers (0 Falls): cadence (steps/min) ↓, walking speed (m/s) →, stride length (m) ↑, stride time (s), single-support time (s) 2. Fallers (1 or more falls): cadence (steps/min) ↓, walking speed (m/s) ↓, stride length (m) ↓
Toulotte et al., 2006b [84]	walking + motor task (carrying a glass)	manual	VICON 370 system (Oxford Metrics), Three AMTI force plates (250 Hz), 10 (m)	cadence (steps/min), walking speed (m/s), stride length (cm), step length (cm), single support time (s)	1. Non-Fallers (0 Falls): cadence (steps/min) ↓, walking speed (m/s) ↓, stride length (cm) ↓, step length (cm) ↑, stride time (s) ↑, step time (s) ↓, single-support time (s) ↓ 2. Fallers (1 or more falls): cadence (steps/min) ↓, walking speed (m/s) ↓, stride length (cm) ↓, step length (cm) ↓, stride time (s) ↑, step time (s) ↓, single-support time (s) ↑ Significant difference (*P* < 0.05) between the fallers and non-fallers under DT-conditions for cadence, walking speed, stride time, step time and single-support time.
Verghese et al., 2017 [85]	walking + word task (reciting alternate letters of the alphabet)	auditory -verbal	electronic walkway (Zenometrics LLC, Peekskill, NY), 14 (ft)	cadence (steps/min), walking speed (m/s), stride length (cm), step length (cm), double support time (%) stride time (s), step time (s)	1. Non-fallers (0 falls) and Non-multiple fallers (0 or 1 fall) Fallers (1 or more falls), Multiple fallers (2 or more falls):cadence (steps/min) ↓, walking speed (m/s) ↓, stride length (cm) ↓, step length (cm) ↓, stride time (s), step time (s), double support time (%) ↑
Asai et al., 2014 [87]	1. walking + arithmetic task (counting backwards from 100 by subtracting serials 1 s) 2. walking + motor task (carrying a ball on a tray)	1. auditory- verbal 2. auditory- motor	triaxial accelerometer, 20(m)	walking speed (m/s) STV (%), RMS in the ML direction (m/s^2^), RMS in the AP direction (m/s^2^), Standardized RMS in the ML direction (%), Standardized RMS in the AP direction (%)	1. no-FOF and FOF and arithmetic task: walking speed (m/s) ↓, STV (%) ↑, Standardized RMS in the ML direction (%) ↑, Standardized RMS in the AP direction (%) ↑ 1b.no-FOF and FOF and motor task: walking speed (m/s) ↓, STV (%) ↑, Standardized RMS in the ML direction (%) ↓, Standardized RMS in the AP direction (%) ↓ Subjects with FoF walked slower during cognitive-task gait than subjects without FoF and walked with greater STV during single-task gait than subjects without.
Donoghue et al., 2013 [88]	walking + verbal fluency task (recite alternate letters of the alphabet (A-C-E, etc.))	auditory- verbal	GAITRite, 4.88 (m)	gait speed (m/s), stride length (m), Double support phase (%), stride length CV(%), stride time CV (%), step width (cm)	1. no-FOF, FOF-NAR, FOF-AR: gait speed (m/s) ↓, stride length (m) ↓, Double support phase (%) ↑, stride length CV (%) ↑, stride time CV (%) ↑, step width (cm)↑ FOF-NAR and FOF-AR groups significantly different to no-FOF group in gait speed (*p* > 0.001), stride length (*p* > 0.001), an DSP (*p* > 0.001).
Reelick et al., 2009 [89]	1. walking +arithmetic task (counting backwards by subtracting serial 7 s) 2. walking +verbal fluency task	1. auditory- verbal 2. auditory- motor	GAITRite, Balance during walking(SwayStar), 10 (m)	gait velocity (cm/s) Stride-length variability (% CV), Stride-time variability (% CV), Mediolateral angular displacement (deg.), Mediolateral angular velocity (deg./s	1. no-FOF and FOF and arithmetic task: gait velocity (cm/s) ↓, Stride-length variability (% CV) ↑, Stride-time variabilit (% CV) ↑, Mediolateral angular displacement (deg.) ↑, Mediolateral angular velocity (deg./s) ↑ 2. no-FOF and FOF and verbal fluency task: gait velocity (cm/s) ↓, Stride-length variability (% CV) ↑, Stride-time variability (% CV) ↑, Mediolateral angular displacement (deg.) ↑, Mediolateral angular velocity (deg./s) ↑ Significantly lower gait velocity for walking at the preferred velocity and during the performance of both dual tasks in the FoF group compared to the no-FoF group. Stride-length and stride time variability were significantly higher in the FoF group dur.ing DT Stride-time variability was also significantly higher in the FoF group when walking at the preferred gait velocity and while performing the arithmetic task.
Wollesen et al., 2017 [90]	walking + visual Stroop task (recite colours, not words)	auditory- verbal	treadmill (h/p/cosmos, Zebris; Isny, Germany: FDM-T)	step length (cm) step width (cm), and gait line (mm)	Intervention with FES-I < 20: and FES-I > 20 step length (cm) ↑, step width (cm) ↓, and gait line (mm) ↑ Control group with FES-I < 20: step length (cm) ↓, step width (cm) ↑, and gait line (mm) ↑ Control group with FES-I > 20: step length (cm) ↑, step width (cm) ↑, and gait line (mm) ↑*:*

Legend: *FOF* Fear of falling, *FOF-NAR* Fear of falling with no activity restriction, *FOF-AR* Fear of falling with activity restriction, *CoF* Concern of falling, *DSP* Double support phase, *CV* Coefficient of variation, *COP* Center of pressure, *COP-Vel* Center of pressure velocity, *ML* Medio-lateral direction, *AP* Anterior-posterior direction

### Statistical analysis of the meta-analysis

For each of the outcome variables of interest (gait speed, cadence, stride length, step length; see Table 2) we collected the gait data for single and dual-task performance. The gait data was presented as differences in means (MD), since the outcome measurements were made or could be converted on the same scale (e.g., meters per seconds). Most of the studies reported means and SDs permitting effect size estimation, otherwise, they were derived from other summary statistics reported in the articles, such as *t*-values or *p*-values. The gait data from individual studies were then pooled in meta-analyses to estimate the overall effect of cognitive-motor interference of gait. Studies were grouped by cognitive task domain and individual meta-analyses were conducted for each outcome: gait speed, cadence, stride length and step length.

In order to determine whether studies shared the same overall effect size or whether the overall effect for a given outcome was modified by certain factors, we conducted a subgroup analyses on studies that directly compared two factors of interest (e.g., arithmetic task vs. verbal fluency tasks) or two groups of participants (e.g., fallers vs. non-fallers) within the same study. Subgroup analyses were conducted using a mixed-effects model and the summary effects within subgroups were computed using a random-effects model. Moreover, to further analyse the differences between fallers and non-fallers as well as participants with and without CoF, DTC were calculated by subtracting the DT values from the ST values. A random-effects model with a generic inverse variance method was used in the pooled analyses, which gives more weight to studies with less variance. Results are presented as effect size with 95% confidence interval (CI) and respective values for null hypothesis tests (e.g., cognitive-motor interference has no effect on gait). Heterogeneity between studies was investigated by calculating the Q-value and I^2^ statistic which quantified the proportion variation that is due to heterogeneity rather than chance. Quantitative syntheses and meta-analyses were produced using Review Manager 5 Software (RevMan 5).

## Results

**Fig. 1:**
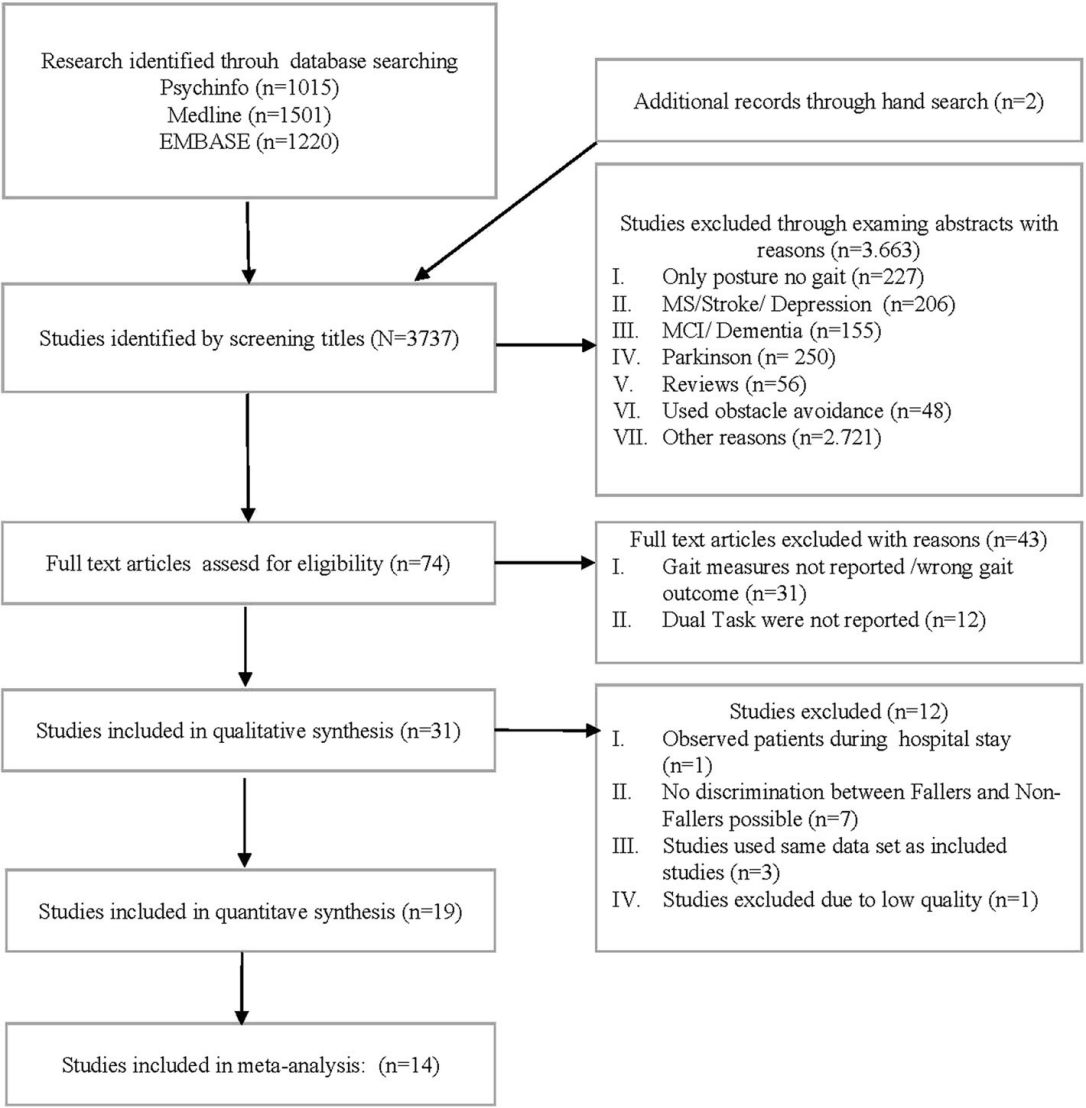
Flow chart of the systematic review procedure

Databases and references identified 2,670 unique articles for consideration. After abstract consideration and title screening, a total of 71 studies were included for further consideration. Reasons for exclusion were studies using participants with neurological disease (e.g., Multiple Sclerosis, Stroke), studies using obstacle negotiation or Reviews. After applying the inclusion criteria, 19 studies were assessed for quality and 16 papers were included in the meta-analysis (cf. Fig. 1; for excluded studies cf. Table 6 and Table 7).

**Table 6:** Excluded paper

Excluded papers	Reason for exclusion
Beauchet, O., Allali, G., Annweiler, C., Berrut, G., Maarouf, N., Herrmann, F. R., & Dubost, V. (2008). Does change in gait while counting backward predict the occurrence of a first fall in older adults? *Gerontology, 54*(4), 217–223. [96]	Similar study with the same data set included in the study
Faulkner, K. A., Redfern, M. S., Cauley, J. A., Landsittel, D. P., Studenski, S. A., Rosano, C., et al. (2007). Multitasking: Association between poorer performance and a history of recurrent falls: Association between poorer performance and a history of recurrent falls. *Journal of the American Geriatrics Society, 55*(4), 570–576. [10]	No discrimination between Fallers and Non-Fallers possible for ST and DT gait data
Kressig, R. W., Herrmann, F. R., Grandjean, R., Michel, J. P., & Beauchet, O. (2008). Gait variability while dual-tasking: Fall predictor in older inpatients? *Aging Clinical & Experimental Research, 20*(2), 123–130. [97]	Observed Inpations and only falls in hospital
Hadjistavropoulos, T., Carleton, R. N., Delbaere, K., Barden, J., Zwakhalen, S., Fitzgerald, B., et al. (2012). The relationship of fear of falling and balance confidence with balance and dual tasking performance. *Psychology & Aging, 27*(1), 1–13. [98]	No discrimination between Fallers and Non-Fallers possible
Halvarsson, A., Oddsson, L., Olsson, E., Faren, E., Pettersson, A., & Stahle, A. (2011). Effects of new, individually adjusted, progressive balance group training for elderly people with fear of falling and tend to fall: A randomized controlled trial. [Erratum appears in Clin Rehabil. 2012 Nov;26(11):1055 Note: Oddsson, Lars [added]]. *Clinical Rehabilitation, 25*(11), 1021–1031. [99]	No discrimination between Fallers and Non-Fallers possible
Halvarsson, A., Franzén, E., Farén, E., Olsson, E., Oddsson, L., & Ståhle, A. (2013). Long-term effects of new progressive group balance training for elderly people with increased risk of falling - a randomized controlled trial. *Clinical Rehabilitation, 27*(5), 450–458. [100]	No discrimination between Fallers and Non-Fallers possible
Herman, T., Mirelman, A., Giladi, N., Schweiger, A., & Hausdorff, J. M. (2010). Executive Control Deficits as a Prodrome to Falls in Healthy Older Adults: A Prospective Study Linking Thinking, Walking, and Falling. *The Journals of Gerontology Series A: Biological Sciences and Medical Sciences, 65A*(10), 1086–1092. [101]	Similar study with the same data set included in the study
MacAulay, R. K., Allaire, T. D., Brouillette, R. M., Foil, H. C., Bruce-Keller, A. J., Han, H., et al. (2015). Longitudinal assessment of neuropsychological and temporal/spatial gait characteristics of elderly fallers: Taking it all in stride. *Frontiers in Aging Neuroscience, 7 2015*. [102]	No mean and SD discrimination between Fallers and Non-Fallers possible
Rinaldi, N. M., & Moraes, R. (2016). Older adults with history of falls are unable to perform walking and prehension movements simultaneously. *Neuroscience, 316*, 249–260. [103]	No discrimination between Fallers and Non-Fallers possible
Rogan S., Taeymans J., Bangerter C., Simon S., Terrier P., Hilfiker R. (2019). Einfluss von Einfach- und Doppelaufgaben auf Gangstabilitat und Ganggeschwindigkeit bei alteren Menschen: Eine explorative Studie, Influence of single and dual tasks on gait stability and gait speed in the elderly: An explorative study. *Zeitschrift fur Gerontologie und Geriatrie*.52, (1), 23–27 [104]	No discrimination between Fallers and Non-Fallers possible
Yamada, M., Aoyama, T., Nakamura, M., Tanaka, B., Nagai, K., Tatematsu, N., et al. (2011). The reliability and preliminary validity of game-based fall risk assessment in community-dwelling older adults. *Geriatric Nursing, 32*(3), 188–194. [86]	Low Quality score
Yogev, G., Plotnik, M., Peretz, C., Giladi, N., & Hausdorff, J. M. (2007). Gait asymmetry in patients with Parkinson’s disease and elderly fallers: When does the bilateral coordination of gait require attention? *Experimental Brain Research, 177*(3), 336–346. [105]	Similar study with the same data set included in the study

**Table 7:** Excluded paper meta-analysis

Excluded papers	Reason for exclusion
Bootsma-van, d., Gussekloo, J., de, C., van, E., Bloem, B. R., & Westendorp, R. G. (2003). Walking and talking as predictors of falls in the general population: The Leiden 85-Plus Study. *Journal of the American Geriatrics Society, 51*(10), 1466–1471.	No Means and SD (only Median and IQR)
Johansson, J., Nordström, A., & Nordström, P. (2016). Greater Fall Risk in Elderly Women Than in Men Is Associated With Increased Gait Variability During Multitasking. *Journal of the American Medical Directors Association, 17*(6), 535–540.	No mean and SD discrimination between Fallers and Non-Fallers possible
Reelick, M. F., Kessels, R. P., Faes, M. C., Weerdesteyn, V., Esselink, R. A., & Rikkert, M. G. O. (2011). Increased intra-individual variability in stride length and reaction time in recurrent older fallers. *Aging clinical and experimental research*, *23*(5–6), 393–399.LL	No mean and SD discrimination between Fallers and Non-Fallers possible

Thirteen studies showed high quality scores (> 16) and seven studies were of good quality (according to [71]). The study by Yamada et al. [86] was excluded due to a quality score < 10. Table 4 gives an overview of all included studies addressing the comparison of fallers vs. non-fallers and participants with and without concerns about falling. The study by Wollesen et al. [90] could not be integrated into the meta-analysis because they used a fixed gait speed in their measurement design.

### Fallers vs. non-fallers

#### Description of the included studies comparing fallers and non- fallers (*N* = 15)

The mean age of the study population was between 67 years [21, 84, 85] and 87 years [19]. The sample sizes of the studies varied between *N* = 16 [84, 85] and *N* = 1350 [78].

Five studies included a prospective design [19, 74, 76, 77, 85].

The included studies used the following dual-task settings:

- *Arithmetic tasks*: *n* = 7 studies used counting backward tasks [19, 20, 74, 75, 80–82], conducted as counting in steps of one (*n* = 3), three (*n* = 3) or seven (*n* = 3) (cf. Table 3).
- *Verbal fluency tasks*: *n* = 7 studies used verbal fluency tasks [20, 21, 75–77, 80, 81]
- *Motor tasks*: *n* = 5 studies used a motor task [20, 21, 80, 83, 85]
- *Other tasks*: visuo-spatial task [20], Stroop task [20], listening and memory task [82] and reciting of letters of the alphabet [85].
- A total number of six studies analysed more than one task [20, 21, 75, 80–82].

Overall, the studies comparing fallers and non-fallers examined 32 different gait quality variables. Gait speed or velocity was assessed by *n* = 14 studies [19–21, 74, 75, 77–85]. Other gait measures included duration to walk a defined distance (*n* = 2) [19, 77], step length (*n* = 3) [21, 80, 85], stride length (*n* = 4) [14, 83–85], cadence (*n* = 6) [19, 21, 77, 83–85], step time (*n* = 3) [80, 83, 85], stride time (*n* = 5) [21, 77, 81, 83, 85] and double support time (*n* = 3) [77, 80, 85]. Several studies used gait parameters of variability (*n* = 14; eg.: stride time variability (*n* = 3), gait speed variability (*n* = 2) and swing time variability (*n* = 2)). In addition, some studies focused on Center of pressure (CoP) or Center of mass (CoM) displacements, or mechanical power in anterior (AP) and medio-lateral (ML) direction during gait cycles. These outcomes were not included into the meta-analysis because of lack of consistency in calculation methods among studies or infrequent use. To measure gait characteristics, a stopwatch (*n* = 6; from 10 m up to 30 m distance), the GAITrite rite system or another electronic walkway (*n* = 8; from 8 m up to 12 m), camera systems (e.g., Vicon *n* = 3) or insoles (e.g., F-Scan *n* = 3) were used.

**Fig. 2:**
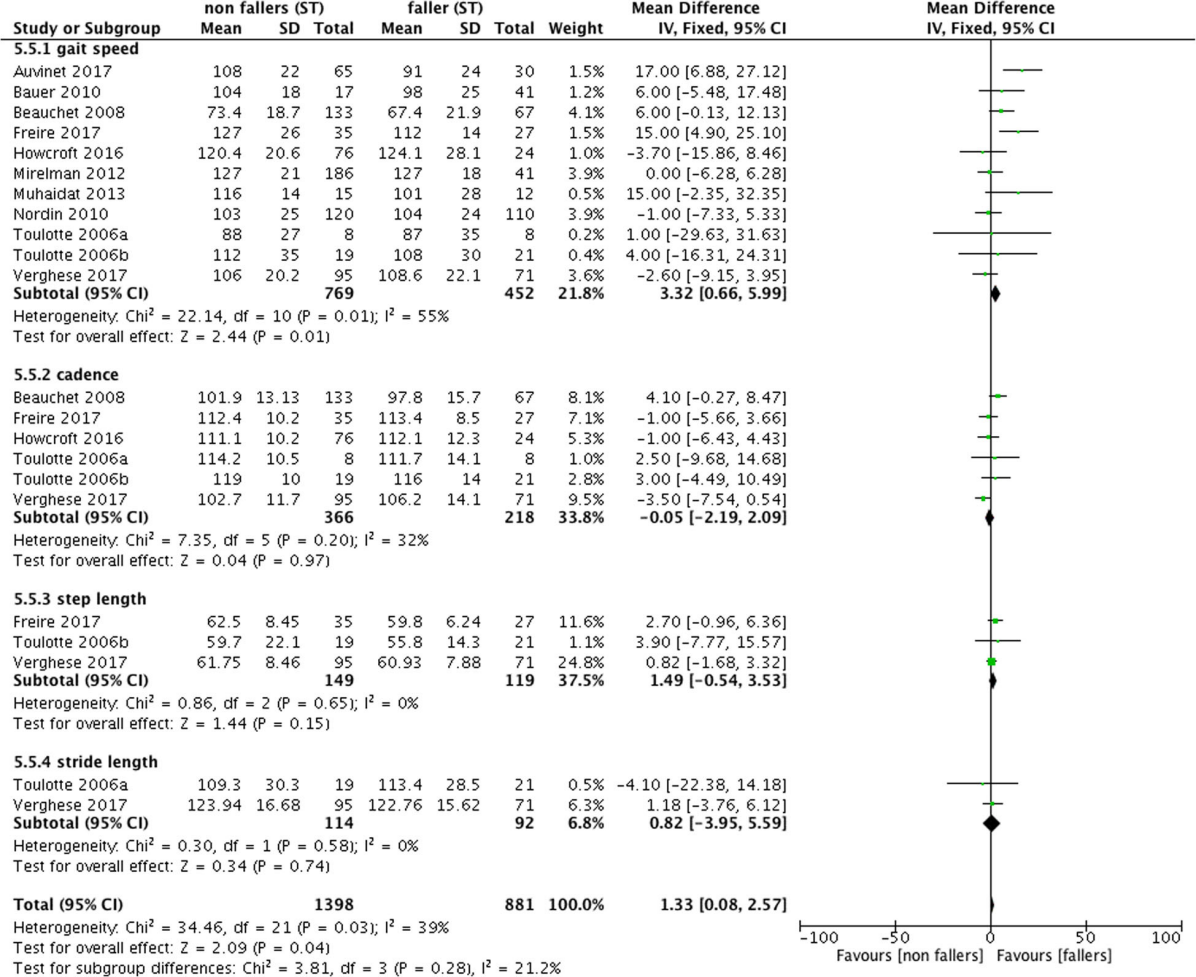
Forest plot meta-analysis of ST performance between non-fallers and fallers

#### Differences on cognitive-motor dual task performance between non-fallers and fallers

Four studies could not be integrated into the meta-analysis because the mean values and SD for the analysed gait data were not reported in comparison of non-fallers and fallers and unavailable after attempting to contact the authors [76, 78, 81]. Independent of the task settings, there were no differences of the gait decrements under DT conditions between fallers and non-fallers (cf. Table 5). Mostly, fallers showed reduced performance of the spatiotemporal gait parameters in comparison to non-fallers. Only two studies used a coefficient of variation [81, 82] and revealed significant differences between fallers and non-fallers with increased variation in fallers. Reelick [81] found a significantly reduced walking performance for the verbal fluency task in comparison to the arithmetic task. Nordin et al. [80] also revealed differences for their task conditions; gait speed increased for the motor-tasks (carrying a cup or a tray) and gait speed decreased for the cognitive conditions (verbal fluency and counting backwards) fallers compared to non-fallers.

#### Results of the meta-analysis fallers vs. non-fallers

**Fig. 3:**
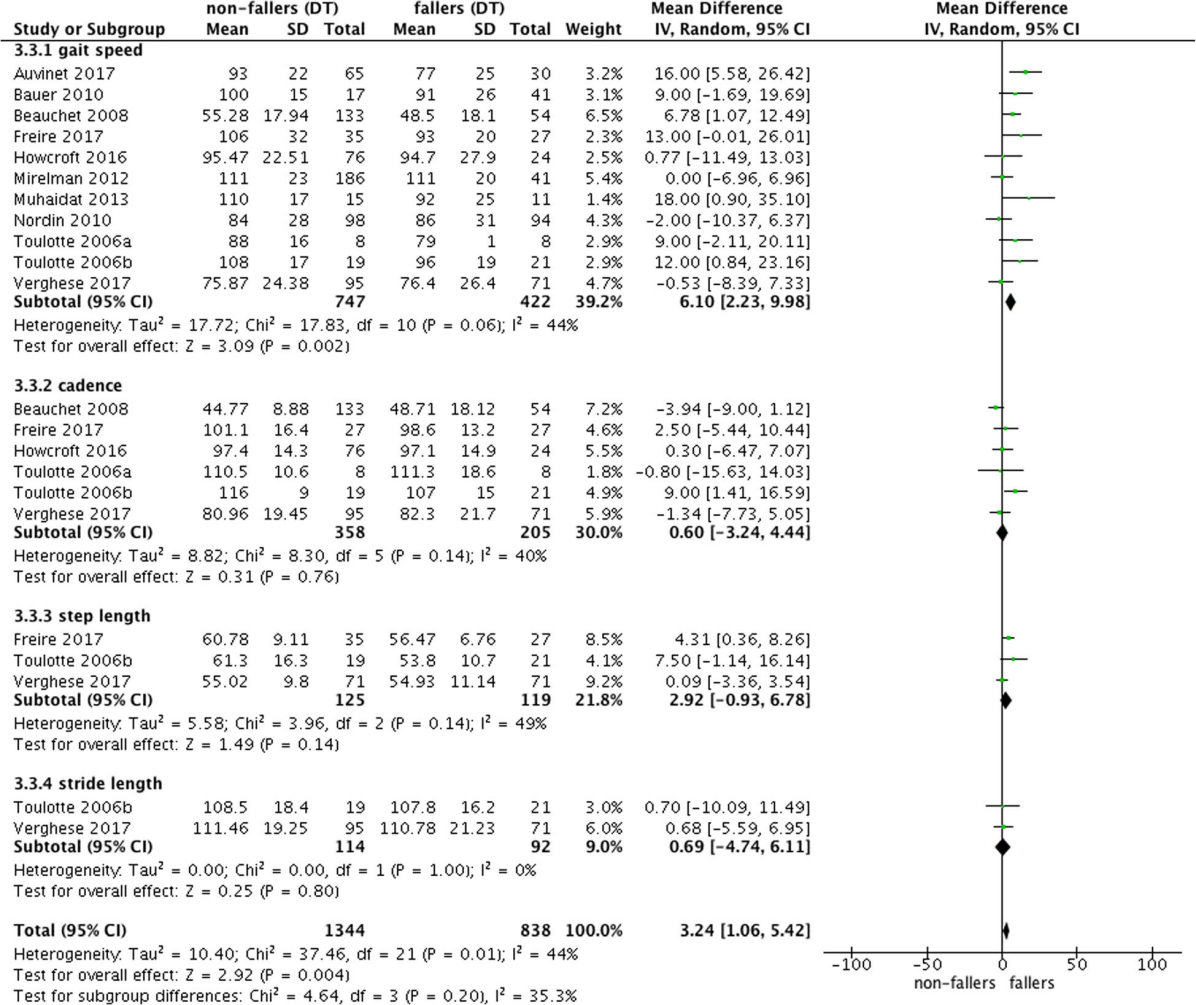
Forest plot meta-analysis of dual-task effect on gait different gait measurement between non-fallers and fallers

The forest plot of Fig. 2 shows significant mean difference of 3.32 [95% confidence interval 0.66–5.99] between non-fallers and fallers for ST gait speed with reduced performance for fallers. However, these results were heterogeneous (I^2^ = 39%; cf. Fig. 2). There were no effects for step length or stride length. Under DT conditions, fallers had a reduced gait speed in comparison to non-fallers with a mean difference of 6.10 [2.23–9.98] (I^2^ = 44%; cf. Fig. 3).

**Fig. 4:**
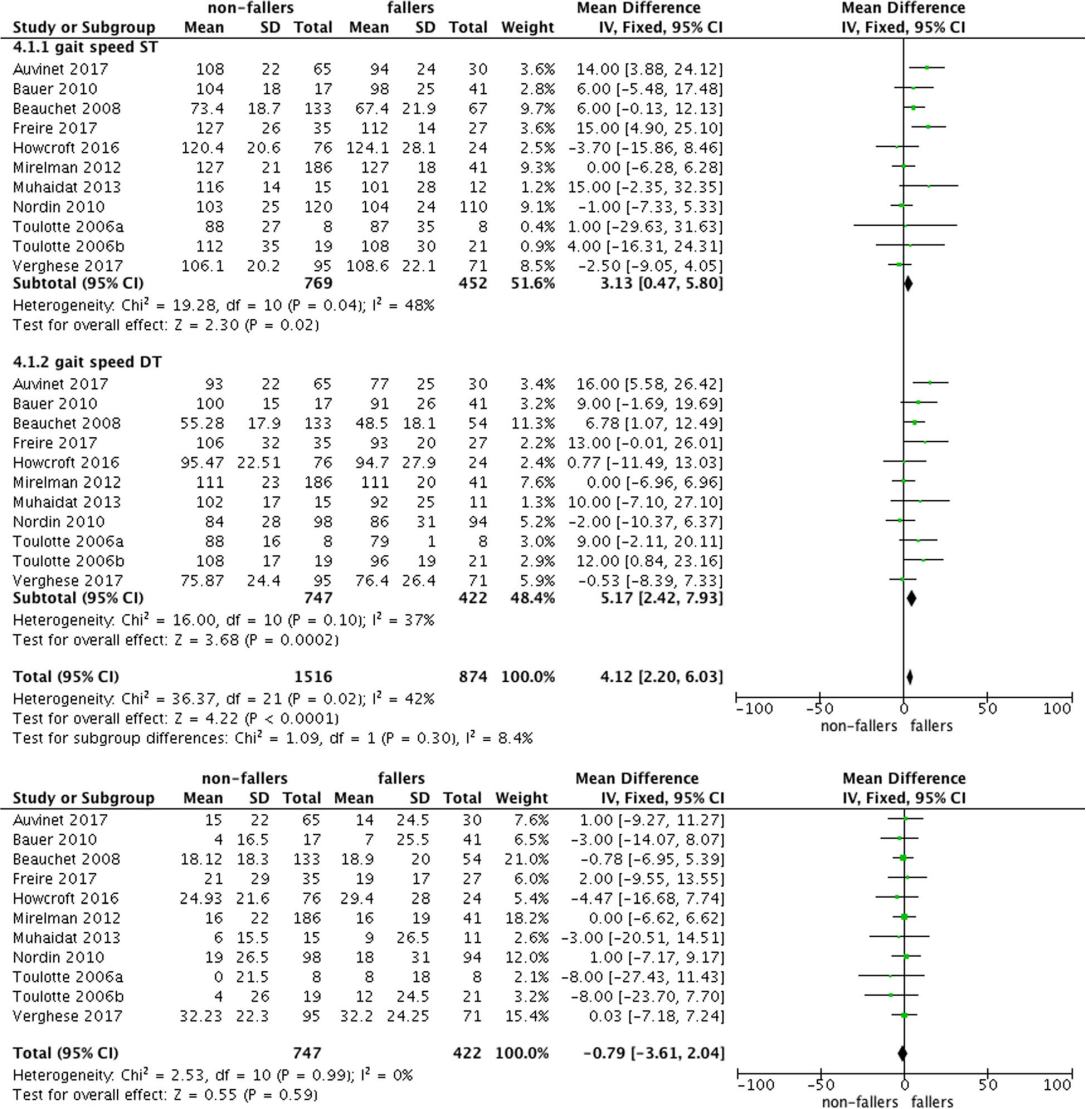
Comparisons of ST and DT gait speed and resulting dual task costs (DTC)

Figure 4 repeats the findings for gait speed under ST and DT conditions and shows the mean difference in DTC (defined as DT minus ST). The meta-analysis showed that there were higher decrements in gait speed for fallers in comparison to non-fallers under DT conditions. However, if the DTC were calculated (Fig. 4), there were no reduced DTC observed for non-fallers.

**Fig. 5:**
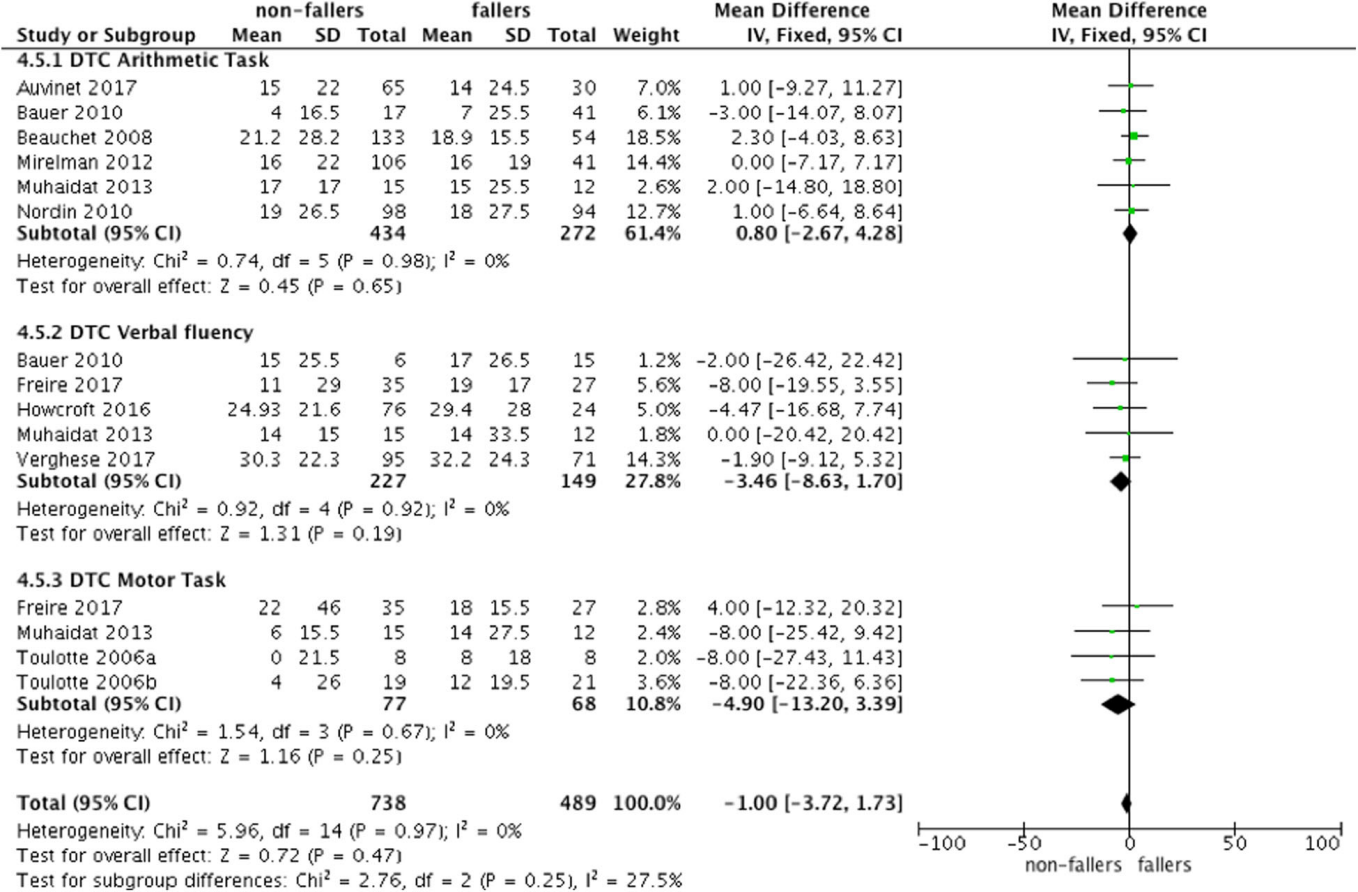
Comparisons of ST and DT and resulting DTC for the different task conditions

Figure 5 visualizes the DTC for the different cognitive task domains. Increased DTC for fallers compared to non-fallers could only be observed for verbal fluency and motor dual-tasks but failed to be significant. The overall effect of the different task conditions was also not significant.

### Participants with concerns about falling vs no concerns about falling

#### Description of the includes studies (*N* = 4) comparing participants with CoF

The mean age of the study population was 69.8 years [90] up to 80.6 years [89]. Sample sizes varied between *N* = 85 [90] and *N* = 1307 [88]. The included studies used different dual-task settings:

- *Arithmetic tasks*: The study by Reelick [100] used a counting backward tasks (subtracting 7 s) and the study by Asai [87] used a counting backward task (subtracting 1 s) (cf. Table 4).
- *Verbal fluency tasks*: Donoghue et al. [88] (recite alternative letters of the alphabet) and Reelick et al. [89] (naming animal species as much as possible) used a verbal fluency task.
- *Other tasks*: The RCT by Wollesen et al. [90] was conducted with a visual-verbal Stroop task.

Studies comparing participants with and without CoF examined 16 different gait variables (cf. Table 5); i.e. gait speed (*n* = 3), stride time variability (*n* = 1), step width (*n* = 2), step length (*n* = 1), stride length (*n* = 2). Two studies used different variability calculations (*n* = 2). Moreover, two studies [87, 89] focused on CoP or CoM displacements in AP and ML direction during gait cycles. To measure gait performance, the GAITrite system or another electronic walkway (*n* = 2; from 5 m up to 10 m), a triaxial accelerometer (*n* = 1) or a treadmill (*n* = 1) were used (cf. Table 5).

#### Differences on cognitive-motor-performance between participants with and without concerns about falling

**Fig. 6:**
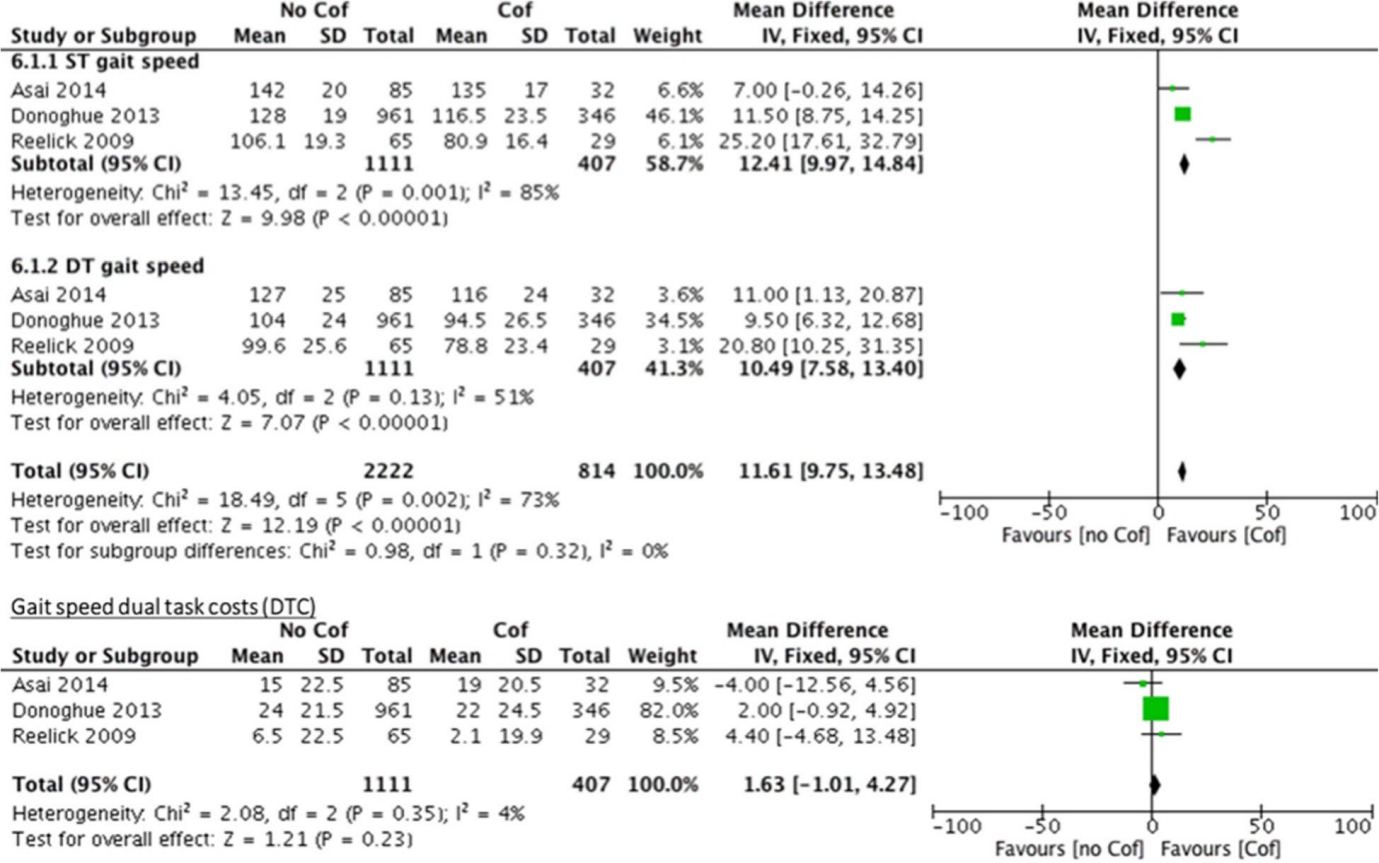
Comparisons of ST and DT and resulting DTC for participants with and without concerns about falling

As reported in Table 5 participants with and without CoF showed comparable DTC. Moreover, all studies showed that participants with CoF had a poorer walking quality (e.g., reduced walking speed with accompanying step length or increased variability) in the ST condition compared to people without CoF. With regard to the different task settings, the two studies that examined two different cognitive dual-tasks found different reactions in all participants according to the task. The study of Asai et al. [87] analysed an arithmetic DT situation and a motor-motor DT situation; and found that both tasks resulted in reduced walking speed. The motor-motor DT resulted in reduced (and therefore improved) body sway in ML and AP direction in comparison to the arithmetic DT situation. Reelick et al. [90] investigated an arithmetic DT situation and a verbal fluency task, and found no task differences. The meta-analysis revealed a significant difference of gait speed between participants with and without CoF under ST (mean difference: 12.41 [9.97–14.84]) and DT (mean difference: 10.61 [7.58–13.40]) conditions. The differences for the DTC failed to show significance (mean difference: 1.63 [− 1.01–4.27]; cf. Fig. 6).

## Discussion

The aim of this systematic review and meta-analysis was to provide a taxonomy of different dual-task settings and test their relations to cognitive-motor decrements with fall risk and CoF. Additionally, the cognitive tasks were regarded separately with the purpose to find a dual-task taxonomy or classification of the DT settings that are most beneficial to identify cognitive-motor interference in older fallers and older people with CoF.

### Differences of DT performance on spatiotemporal gait parameters between non-fallers and fallers

The results of the meta-analysis suggested that gait speed and cadence in ST and DT conditions can discriminate between fallers and non-fallers. Studies classifying people as fallers and non-fallers were based primarily on retrospective falls, with only two studies being prospective [19, 96]. These results confirm previous systematic review evidence which showed differences in gait speed between fallers and non-fallers [50, 68]. With regard to the associated DTC, only five of eleven studies found higher decrements in gait speed from ST to DT for fallers in comparison to non-fallers (Fig. 4). The overall DTC failed to be significant between these two groups in our meta-analysis. There were only small amounts of DTC for both groups and the standard deviations were large. In line with the results of other studies that could not be included in the meta-analysis, fallers and non-fallers both show decrements in gait speed in ST and DT conditions (cf. Table 5 and Fig. 4). These decrements are not significantly different between groups which is inconsistent with the hypothesis that non-fallers and fallers differ in their ability of task prioritization [16, 67]. Fallers walk significantly slower than non-fallers in ST conditions; however, step length and stride length, which are known to be highly correlated with gait speed [91], did not differ significantly between groups. Specific recommendations on whether or not cognitive-motor-interference increases fall risk cannot be provided. These results confirm the findings by Zijlstra et al. [68] and Menant et al. [50] who also reported no additional benefit of DT walking as a measurement to discriminate fallers from non-fallers. Nevertheless, it is important to note that gait performance includes different components of functional performance such as maximal walking velocity, gait economy, walking effectiveness, efficiency and safety. These aspects might be more relevant to estimate fall risk. Therefore, future studies should address these components of gait performance in tailored DT settings.

### Differences of DT performance between participants with and without CoF

People with CoF showed greater gait decrements under ST and DT conditions compared to people without CoF. The overall effects from the meta-analysis suggested that the effects of CoF were larger (11.61; CI: 9.75–13.48) in fallers compared to non-fallers (4.12; CI: 2.20–6.03). CoF is common in people with and without a previous fall history and the prevalence rates are higher than falls themselves [93]. It has been suggested that people with CoF have difficulty inhibiting or ignoring irrelevant information of the environment when controlling their balance in complex and DT situations [40]. Many daily life activities include some level of dual-tasking in which executive functioning or performance (i.e. inhibition) are required. CoF might compete for these limited resources of attentional focus to maintain their balance [52], which would result in a more pronounced slowing of their walking speed under DT conditions (cf. Fig. 6) in people with CoF irrespective of their fall history or fall risk. However, our analyses were not able to confirm this hypothesis as DTC was not significantly different between people with and without CoF.

### Influence of the task condition

A large variety of cognitive tasks have been used to assess cognitive-motor interference in the literature. As part of this review, a total of 11 different DT-conditions were used to compare non-fallers and fallers on DT walking performance (Fig. 5). According to the proposed taxonomy (Table 1) mental tracking tasks, especially counting backward tasks by numbers in 1 s, 3 s or 7 s are the most commonly used task sets. Overall, we were able to compare three types of cognitive dual-tasks (i.e. arithmetic, verbal fluency and motor tasks) within the meta-analysis of this review. Two of them belong to the same category of our taxonomy (mental tracking, cf. Table 1). The third one included an additional motor task. However, all task settings affected DTC similarly, and the pooled effect (mean difference: − 1.00 [− 3.72–1.73]) had low heterogeneity (I^2^ = 0%).

Other cognitive tasks such as reaction time and decision making tasks for processing speed and controlled processing tasks, [92] were not integrated in the task setting of the included studies but could be relevant for navigating in daily traffic situations. In addition, previous studies have suggested that more complex tasks such as working memory tasks, discrimination tasks or visuospatial tasks would have a greater impact on the DTC (for an overview see Lacour et al. [52]) but this could not be confirmed by this review due to the limited studies using these tasks. Furthermore, within the available data there were also no marked differences between the different types of cognitive tasks. On the other hand, there is evidence that mental tracking tasks like verbal fluency tasks increase the DTC more significantly for fallers compared to non-fallers [81], due to the additional load on the working memory for these tasks. However, this review was not able to confirm this hypothesis. Finally, motor-motor DT condition also did not show significant differences in DTC between non-fallers and fallers. Both studies by Toulotte et al. [83, 84] suggested a more pronounced DTC when carrying in glass of water, suggesting this would slow participants down as they need to observe the glass of water in their hand. However, other studies have suggested the opposite [80], as a result of a forward flexion of the trunk when carrying a tray with a glass of water in front of the body.

### Implications of the results

Similar to previous reviews, we were not able to confirm differences between fallers and non-fallers in DTC. One reason for this result might be that we were only able to compare three types of dual-task settings (i.e. arithmetic, verbal fluency and motor tasks) within the meta-analysis. Therefore, additional studies are required to examine the discriminatory ability of walking performance with and without concurrent reaction time, controlled processing, visuospatial, working memory and discrimination tasks. Study designs comparing different DT-settings in smaller samples [20] or randomised trials with a representative larger sample size could be used to systematically address different cognitive processes and their complexities. In addition, it might be important to consider an individual’s biography before deciding on a DT. One might argue, that a maths teacher might find a counting backwards task more intuitive, while a librarian might be more comfortable with verbal fluency tasks. More work is required to test this hypothesis. Tasks that include visuo-spatial information processing or higher executive functions (e.g., inhibition within a Stroop-task) [2] might have greater potential in discriminating between fallers and non-fallers. These tasks may be less dependent on people’s biography. However, these task-settings might be difficult to use in clinical settings and with short walking distances. In addition to the cognitive dimensions of the task settings, the walking conditions and parcourse need to be reflected, as a straight walking course does not sufficiently address real-life gait. The ongoing development of wearable technology might be one solution to overcome measurement set up problems.

### Limitations

Overall, the quality of the included studies was good. Nevertheless, there are some issues that need to be discussed. First, spatiotemporal gait parameters were assessed using diverse measurement methods, varying between the crude use of a stopwatch to accelerometers and electronic walkways [94]. Second, there is not a common length of the walking tracks with many studies using distances that are too short to see a DT effect. According to Lindemann et al. [95], the distance to achieve a steady walking state increases with higher gait speed. Third, studies report different spatiotemporal gait parameters. Especially, spatiotemporal gait parameters related to balance, such as step width, double support time, gait stability and variability, were not reported frequently enough to be included in the meta-analysis. It is possible that the effect of DTC would be visible on such measures before it affects gait speed especially over short distances. Fourth, the short distances might influence prioritisation of the motor and cognitive tasks. The short distances also limit the time available for the cognitive dual-task, which might explain why the meta-analysis could not show a different cognitive-motor interference on gait between fallers and non-fallers. Finally, most of the studies did not report the motor and the cognitive DTC. This means that there is no control for the attentional focus of the participants, rendering it unclear if the performance decrements result from the attentional focus or from cognitive-motor interferences. Finally, to gain information about the influence of the DT taxonomy on DTC, this review integrated only studies with straight walking. This was necessary to overcome the problem that gait execution while changing directions, walking in curves or reacting to external perturbation, has a different impact on spatiotemporal gait parameters as well on the cognitive performance.

## Conclusions

Overall, the large diversity of studies and types of cognitive dual-tasks do not allow us to provide conclusive recommendations for clinical testing of cognitive-motor interference while walking. In agreement with previous studies [50, 78], we found no additional benefit of DT gait analysis to differentiate between fallers and non-fallers. Similar results were found when comparing people with and without CoF. However, our analyses also reveal that several domains of cognitive dual-tasks have not yet been investigated. The proposed cognitive task taxonomy will assist in systematic assessment of these tasks and their effect on gait.

## Data Availability

The supporting data is available via the corresponding author.

## Appendix

**Table 8:** Search strategy

Keywords	Papers identified		
	Medline	EMBASE	PsycInfo
1. "Age" or "old$" or "elder$" or "aged" or "advanced age" or "senior$" or "geriatric$" or "eldest" or "aging" or "gerontic" or "faller$" or "fear of falling"	10.451.411	7.016.103	1.097.778
2. "corresponding task$" or "coupled task$" or "dual task$" or "dual task paradigm$" or "secondary task" or "conflicting task" or "task prioritisation" or "inattentional blindness"	4.200	167.739	56.654
3. "gait" or "step length" or "cadence" or "step count" or "step width" or "stance time" or "swing time" or "single support time" or "stride time" or "stride width" or "stride length" or "gait line" or "maximum force forefoot" or "maximum force midfoot" or “maximum force heel” or “double support time” or “gait speed” or “stride speed” or “motion” or “movement$” or “motor$” or “locomotion” or “walking” or “balance” or “posture” not “slipping” (not “perturbation”)	1.198.325	1.374.933	349.516
4. “cognitive” or “neurocognitive” or “cognition$” or “executive” or “processing” or “spatial” or “visuospatial” or “memory” or “reaction$” or “speed” or “decision-making” or “mental” or “attention” or “cognitive-motor” or “motor-cognitive” or “reaction$” or “planning” or inhibition	4.885.498	7.293.833	1.589.107
5. Combination of 1 and 2 and 3 and 4	1.515	658	1.134
